# Impact of supplementary sensory feedback on the control and embodiment in human movement augmentation

**DOI:** 10.1038/s44172-023-00111-1

**Published:** 2023-09-11

**Authors:** Mattia Pinardi, Matthew R. Longo, Domenico Formica, Matija Strbac, Carsten Mehring, Etienne Burdet, Giovanni Di Pino

**Affiliations:** 1 NEXT: Neurophysiology and Neuroengineering of Human-Technology Interaction Research Unit, Università Campus Bio-Medico di Roma, Rome, Italy; 2 Department of Psychological Sciences, Birkbeck, University of London, London, UK; 3 School of Engineering, Newcastle University, Newcastle upon Tyne, UK; 4 Tecnalia Serbia Ltd, Belgrade, Serbia. University of Belgrade-School of Electrical Engineering, Belgrade, Serbia; 5 Bernstein Center and Faculty of Biology, University of Freiburg, Freiburg, Germany; 6 Department of Bioengineering, Imperial College of Science, Technology and Medicine, London, UK

**Keywords:** Biomedical engineering, Physiology

## Abstract

In human movement augmentation, the number of controlled degrees of freedom could be enhanced by the simultaneous and independent use of supernumerary robotic limbs (SRL) and natural ones. However, this poses several challenges, that could be mitigated by encoding and relaying the SRL status. Here, we review the impact of supplementary sensory feedback on the control and embodiment of SRLs. We classify the main feedback features and analyse how they improve control performance. We report the feasibility of pushing body representation beyond natural human morphology and suggest that gradual SRL embodiment could make multisensory incongruencies less disruptive. We also highlight shared computational bases between SRL motor control and embodiment and suggest contextualizing them within the same theoretical framework. Finally, we argue that a shift towards long term experimental paradigms is necessary for successfully integrating motor control and embodiment.

## Abstract (web summary)

Supernumerary robotic limbs are robotic devices providing additional limbs to the user. Mattia Pinardi and colleagues review the impact of supplementary sensory feedback on the control performance and embodiment of supernumerary robotic limbs.

## Introduction

The development and use of tools to overcome environmental challenges is one of the most unique features of the human being; other animals can also make use of tools in creative ways, but how tools shaped human evolution (and vice versa) has no equal in other species^1,2^. Indeed, humans’ greatest chance for survival were the tools their brain could conceive, and just as the brain grew in complexity and power, the same happened to their tools^3,4^.

The introduction of advanced artificial devices to improve physical and cognitive human capabilities^5,6^ can be seen as a modern achievement of tool development. Robotics have been recently developed for able-bodied augmentation^7,8^, amplifying its possible impact and the interest of the scientific community and general public.

Human augmentation can be related to several domains: movement, sensory and even cognitive enhancement^6^. Movement augmentation translates the application of strategies and devices originally designed to compensate for lost functions (e.g., control interfaces and exoskeletons) to healthy people, but exploits also devices such as robotic manipulanda.

This paper focuses on sensory feedback in human movement augmentation (HMA), i.e., providing users with cues about the tool and its interaction with their body and the environment. This is different from sensory augmentation, i.e., empowering human senses to detect information that we are not originally able to sense (e.g., ultrasound). More specifically, we will focus on the impact of sensory feedback on control and embodiment of Supernumerary Robotic Limbs (SRLs), predicting that this will allow higher performance during object manipulation and environmental interaction, thus reshaping how the user represents the tool and their body. This afference-focused approach constitutes a novelty in the field of human augmentation. Indeed, the other recent works that deal with this topic^9–11^ mainly address the challenge of controlling SRLs and overcoming the barriers to human movement augmentation (e.g., allocating limited neural resources). On the other side, we present a complementary discussion that systematically considers the afferent branch of the human-robot closed loop. Additionally, while sensory feedback has been considered previously by other authors, they focused primarily on different contexts, such as prosthetics^12^ and robotics^13,14^, without addressing the feedback-related challenges that are specific to the field of human augmentation as we do in the present work.

Several entries in the newly proposed taxonomy of human augmentation^10^ clearly highlight the main benefits of SRLs: (i) having an additional limb allows to perform tasks beyond what is possible with two hands; (ii) once properly integrated in the human sensorimotor loop^15^, SRLs could augment sensorimotor capabilities of healthy humans, for example increasing the user’s force (power augmentation) or precision; (iii) they allow the user to reach and manipulate objects at more distant spatial locations (workspace augmentation); (iv) they open up new ways to interact with the environment by increasing the number of degrees-of-freedom (i.e., DoF, allowed movements and orientations in space) available to the user (DoF augmentation). Moreover, SRLs could be exploited for a wide range of applications, from handling hazardous materials to health care and to heavy-duty industrial employment. Finally, by studying cortical plasticity after repeated use of SRLs^6^, we can improve our understanding of how human-robot interaction changes cortical body and space representations^16,17^.

Several SRL prototypes are currently employed in laboratory environments, most of them in the form of additional fingers^18–22^ or arms^23–25^. They are usually controlled by tracking movement of other limbs not directly involved in the task^19,26^, by reading user’s postural adjustments to trigger robotic support^27,28^ or by employing dedicated interfaces such as joysticks^20^. While in the future SRLs could be employed in dexterous tasks (i.e., robotic surgery), currently they mostly help users in physical works, minimizing the human effort while bearing a load^24,27^ by leaning against walls and surrounding structures^8^, thus increasing operational safety.

However, to achieve effective human augmentation, it is not sufficient to provide the user with an SRL and the means to control it. Indeed, the vast literature concerning the role of sensory feedback in motor control shows that a closed loop system provides important benefits: it not only improves motor performance^29–32^, but also increases the feeling of embodiment of an artefact^33–36^ and enhances users’ acceptance, as found in prosthetics research^37–39^. Indeed, the ability of a closed loop control to impact the representation of the body and of the space and objects surrounding us^40^ could set human augmentation apart from the simple use of a tool. Incorporating a tool into body representation^41^ and perceiving it as part of our own body (i.e. embodying it), could influence its skilful use and alter natural body kinematics^42,43^. These findings highlight the importance of a seamless integration of afferent (i.e., sensory feedback) and efferent information (i.e., motor commands) relative to the robotic limb, as happens with the natural body.

This work will introduce neuroscientific key concepts for human motor control and embodiment, framing them in the context of HMA (Section “Multisensory Integration and Human Augmentation”), present the impact of sensory feedback on SRL control (Section “Sensory Feedback and SRL Control”) and embodiment (Section “Sensory feedback and SRL embodiment”), and discuss main aspects worth to be considered in future experimental approaches (Section “Discussion and Conclusion”).

## Multisensory integration and human augmentation

Before delving deeper into the relationship between SRL and sensory feedback, it is important to consider how multisensory cues are managed by the brain, since this is a pivotal process for any kind of sensorimotor activity, including those which exploit artificial limbs.

This mechanism, called multisensory integration, likely follows a Bayesian approach to continuously combine afferent streams of multisensory information in a stochastically efficient manner^44,45^. Hence, cues coming from different sensory modalities are weighted according to their reliability: the more reliable and high-fidelity a sensory signal is, the more it contributes to the perceptual experience. This process leads to rich multisensory perception and understanding of the world^46–48^, to the creation of causal relations between events^49^ and to refined motor performance^50,51^. Translating these benefits to HMA, it can be hypothesized that if users could receive multisensory feedback regarding the SRL, such as its configuration, movement and interaction with the environment, they would become more proficient in controlling it, with reduced learning time. Indeed, multisensory information has been shown to prompt motor learning more than unisensory cues^52^.

Multisensory integration has also been shown to be closely linked to our experience of embodiment^40^, which can be boosted by multisensory spatiotemporal congruency^44^. Embodiment is the feeling that our body belongs to us, and despite seeming a simple and immediate concept, it hides a complex and multi-componential nature, which has been a focus of neuroscientific literature for many years^53,54^. The possibility of extending embodiment to objects that are not originally part of our body, such as tools, can have a strong resonance on their users, and more generally, on the research field of HMA. Indeed, the representation of our body and the relationship between its parts, which is tightly related to embodiment, has proven to be surprisingly plastic. By tricking the brain through synchronous tactile stimulation (i.e. brushstrokes) of the real hidden hand and of a rubber hand, it was demonstrated that a fake hand can be perceived as being part of our body, i.e., the rubber hand illusion^55^. Numerous studies replicated these results and extended them in a variety of ways^56–58^, giving birth to a larger family of multisensory illusions of embodiment which includes also full body illusions^59^ and enfacement illusions^60^, to name a few.

Multisensory integration has been also exploited in patients suffering from phantom limb pain^61,62^, who have been treated with mirror therapy, suggesting that visuomotor integration can alter the body schema (i.e., an action oriented body representation)^41,63^. The connection between action and fundamental mechanisms of embodiment, such as body representation, has led scientists to speculate that including an external tool (e.g., an SRL) into the body representation might improve the ability to control it^64,65^. Exploiting a virtuous cycle between embodiment and motor control, based on multisensory integration, would prove particularly useful in human augmentation. Indeed, efficient SRL control is an important challenge to overcome due to the lack of neural resources and pathways that can be repurposed, as it happens with prosthetics^11^ and because, contrarily to robotics, human augmentation requires SRL to be controlled simultaneously with, and independently from, natural limbs.

## Sensory feedback and SRL control

Why sensory feedback is a pressing matter for SRL control can be understood if we consider how motor control works in humans. Sensory feedback informs us about the outcome of our movements, allowing us to adjust motor planning, if necessary, in order to better match the desired outcome^66^. However, relaying and computing sensory feedback introduces a certain delay, making a control based only on this policy relatively slow.

To speed up control and increase the accuracy, motor control strongly relies on prediction and anticipation, leveraging on dynamic representations of the body and the environment, known as internal models^67–70^. These representations are exploited as inverse models to determine the motor command needed to reach a specific body configuration, and as forward model to predict the sensory consequences of an action before it actually takes place, thanks to an efference copy of the motor command. Then a State Estimate is computed by comparing the prediction generated by the forward model and the actual sensory feedback, and it is used to refine the successive motor command^71^.

Skilled control of a tool depends on the correct computation of the limb dynamics and kinematics by the internal models^72^ and this may likely be valid for SRL too. Internal models are acquired and adapted thanks to sensory feedback, and this adaptation improves motor performance, for instance in the control of prostheses^12,73–75^. However, despite the critical impact of feedback on the quality of motor control, most SRL studies have focused on how to send motor commands^19,25,28,76–82^ by relying exclusively on visual or auditory cues (i.e. incidental feedback) for closing the loop, neglecting any kind of unobtrusive supplementary feedback.

Hence, several open questions need to be addressed to implement an efficient SRL feedback, including: which information about the SRL status should be relayed to the user and how? Which tool should be used to relay it? Should the sensory modality between SRL and user be the same (e.g., exert a pressure on user’s body to convey robot contact force) or would a cross-modal stimulation be more convenient in some cases? When is feedback useful, without becoming a cognitive burden? How is SRL feedback going to impact fundamental parameters of motor control (e.g., accuracy or exerted force)?

### Features of the SRL Feedback

For operational purposes, in this review, we label as “content” the physical quantity relative to a part of the SRL which is relayed by the feedback (e.g., the position of the SRL end-effector), and as “parameter” the way to describe the content considering different reference frames (e.g., its distance from the target or from the workspace centre).

Furthermore, “modality” (e.g., touch) and “sub-modality” (e.g., pressure or vibration) are related to the sensory modality and sensory channels exploited to deliver the information extracted from the “source” (i.e., SRL). Finally, the “encoding algorithm” (e.g., stimulation intensity proportional to the distance between end-effector and target position) defines how the parameter is translated into activity of the “stimulating device” (e.g., vibrotactile stimulators), and the “stimulation pattern” defines the spatial and temporal distribution of the delivered stimulation. All these definitions and labels are somewhat fluid and often overlap, depending on the specific design of different studies.

**Fig. 1:**
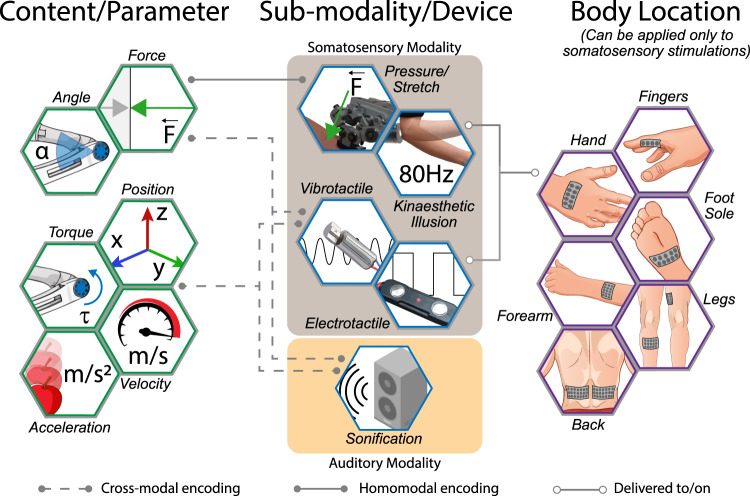
SRL sensory feedback features. Information on the state of the robot (Source, green hexagons) are collected either through sensors embedded in the SRL (e.g. joint torque sensors) or through external dedicated systems (e.g. markers for Cartesian position tracking). SRL status is then encoded into a stimulation pattern which involves a specific sub-modality. The device employed to deliver the stimulation, determines if the encoding is homo-modal (e.g. pressure actuators exert a force on the user’s body proportional to interaction force of the SRL) or cross-modal (e.g. SRL distance from target is translated into a sound). In both cases, the resulting stimulation pattern is relayed to the user as supplementary sensory feedback (blue hexagons). Different user feedback modalities are represented by yellow (auditory) and grey (somatosensory) squares. Supplementary somatosensory feedback can be delivered to specific locations on the participant’s body (purple hexagons).

The first question we address is which content should be retrieved from the source (Fig. 1, left). Kinematic and dynamic contents related to SRL, such as its contact force, position, acceleration, velocity and joint configuration have often been selected for supplementary feedback signal (see Table 1) partly because some of them cannot be easily accessed through vision (e.g., contact force and acceleration), but also because they are fundamental for dexterous movements^83^.

**Table 1:** Studies which investigated the impact of supplementary sensory feedback on the control of an SRL.

Reference	Content	Parameter	Submodality/Location	Encoding algorithm	Pattern	SRL	Impact on control
Guggenheim et al.^122^	Contact Force	Combination of torque generated by the actuator and force exerted by the EE on a surface	Pressure applied against the lower back	Pressure proportional to force intensity	Continuous	Wearable, belt-attached robotic arms	Better force regulation and supervision of SRL*
Saraiji et al.^123^	Contact Force	Pressure recorded on the EE tip	Pressure applied to soles of the feet	Pressure proportional to force intensity	Continuous	Wearable, back-attached anthropomorphic robotic arms	Good performance in pointing task (validation experiment)
Hussain et al.^143^	Contact Force	Pressure recorded on the EE tip	Vibrotactile stimulation to the controlateral finger	Vibration frequency proportional to force intensity or discrete contact signal/threshold	Continuous/ Burst	Wearable sixth robotic finger	Lower completion time and exerted force in a pick-and-place task*
Hussain et al.^119^	Contact Force	Pressure recorded on the EE tip	Vibrotactile stimulation to the contralateral, healthy finger (used in chronic stroke patients)	Vibration frequency proportional to force intensity or discrete threshold signal	Continuous/ Discrete	Wearable sixth robotic finger	Improvement in 2 out of 5 activities of the Frenchay Arm Test, subjective preference for feedback vs no feedback, but no difference in performance*
Aoyama et al.^121^	Joint angle	–	Vibrotactile stimulation to induce a tactile phantom sensation on the contralateral hand	Vibration frequency proportional to joint angle amplitude	Continuous	Wearable robotic extra thumb	Higher number of successful contacts between artificial and natural fingers when receiving feedback
Sobajima et al.^120^	Contact force	Pressure recorded on the EE tip	Electrotactile stimulation on the controlateral thumb	Electrotactile stimulation amplitude proportional to force intensity	Continuous	Wearable robotic extra thumb	Lower completion time and less errors in a bolt picking task*
Segura Meraz et al.^20^	Contact force	Pressure recorded on the EE tip	Electrotactile stimulation on the controlateral thumb	Electrotactile stimulation amplitude proportional to force intensity	Continuous	Wearable sixth robotic finger	Better performance in pointing task*
Hernandez et al.^124^	Contact force	Torque exerted by the actuator	Pressure applied to the feet sides	Pressure proportional to force intensity	Continuous	Non-wearable robotic arms	High force transparency in bipedal grasps and compensation for abrupt disturbances*
Noccaro et al.^84^	EE Position or Joint torques	Distance of EE from centre of workspace or joint torques	Vibrotactile stimulation to ipsilateral leg	Vibration frequency proportional to EE distance from centre/joint torques	Continuous	Non-wearable robotic arm	Lower completion time and higher accuracy in EE offline tracking task using cartesian position vs joint torque feedback
Pinardi et al.^153^	EE Position	Distance of EE from centre of workspace	Vibrotactile stimulation to ipsilateral leg	Vibration frequency proportional to EE distance from centre	Continuous	Non-wearable robotic arm	Higher accuracy compared to catch trials (sham feedback) in EE online tracking task
Pinardi et al.^126^	EE Position or Joint angles	Distance of EE from centre of workspace or joint angles	Vibrotactile stimulation to ipsilateral leg	Vibration frequency proportional to EE distance from centre/joint angles	Continuous	Non-wearable robotic arm	Higher accuracy in EE online tracking task using cartesian position vs joint angle feedback

Empty parameter slots indicate that it was not possible to disentangle parameter from content. Submodality and Location are merged in a single column. '*' in the Impact on Control column indicates studies in which participants had also visual feedback in addition to supplementary feedback, while completing the task.

Despite the attention those haptic contents have received, the systematic investigation of their specific impact on SRL control performance is somewhat lacking. In a recent paper which employed an SRL^84^, two different feedback-encoding paradigms based on kinematic and dynamic approaches were compared, namely Cartesian position and joint torques. It is important to note that, given the focus of the present work, dynamic and kinematic are adopted here as usually defined in robotics, i.e., considering or not the causes of motion, respectively. After a limited training (about 30 min) in a planar, slow-moving task, Cartesian space feedback allowed blindfolded participants to track the position of the robotic end-effector faster and more accurately, compared to joint torque feedback. To the best of our knowledge, no other studies have directly compared the effectiveness of different parameters in describing the posture of the SRL.

However, a study involving blindfolded participants showed that different contents can be proficiently combined into a single feedback signal delivered through vibrotactile stimulators: 80% of position (parameter*:* distance from the workspace centre) and 20% of velocity of the unseen hand obtained the best accuracy and lowest proprioceptive drift in stabilization and reaching tasks^85^. Additionally, the usefulness of the feedback content is heavily influenced by the task it is referred to or even by a specific task phase. Hence, we could speculate that in the case of a mixed content feedback, the percentage of each information (i.e., its weight) could be modified in real time by a smart algorithm depending on the task or activity, easing the burden of optimal weighting on the attention of the user. For example, during a task which combines reaching and grasping or pushing, users could receive a feedback signal heavily influenced by SRL Cartesian position during the first part, but when they finally interact with the environment through the end effector, joint torque or force feedback could become prevalent in the overall feedback signal. However, further investigation on this aspect is required, since the user would most likely require intensive training to be able to discriminate different contents carried by the same signal.

Despite heat and nociceptive stimuli not being strictly and directly involved in motor control, but rather in avoiding specific positions or trajectories^86^, their translation to the user could be worth investigating^87^. No studies have explored this possibility, most likely because of their prevalent use in low level sensorimotor loops not involving the user (e.g., detection of high temperature leads to device shutdown to prevent damage), and the difficulty of establishing analogies between pain and SRL’s parameters. However, heat and pain information collected through dedicated sensors or inferred from already available parameters (e.g., nociceptive cues from excessive forces that could cause a robot’s malfunction) and relayed as non-painful and discreet stimulations could increase environmental awareness, thus potentially improving robot control.

After choosing the supplementary feedback content and its parameter, it is important to carefully select an appropriate user’s sensory modality and sub-modality to deliver the information, which are tightly related to the stimulating device (Fig. 1, centre). While vision is usually a highly reliable sense, vision alone might be non-optimal for conveying SRL supplemental feedback. Indeed, the continuous monitoring of the activity of a SRL through visual attention would imply a considerable cognitive burden, in line with running “a daily marathon”^88^ reported by patients with proprioceptive impairment who rely instead on visual monitoring^89–91^. Additionally, in a real-life scenario vision can be occluded while controlling an SRL by environmental elements (e.g. smoke, dust or debris filling a seismic area) or because of the task itself (e.g. smoke produced by an electrocautery in endoscopic surgery). Finally, being richer, visual information is typically processed more slowly than other modalities^92^. To face this challenge, robotics and prosthetics have focused mainly on haptic feedback^31,93–98^, which also gives the advantage of homogeneity between the sensory modalities of source and user (e.g., deliver the pressure recorded at the end-effector by applying a pressure on users’ body). Also, somatosensation is something we are not constantly aware of during our everyday routine^99^. Considering the context of HMA, this feature becomes particularly interesting because of the lower risk of overloading the attentional cognitive system^100–102^.

We will consider works in the fields of robotics and prosthetics with the final goal of translating findings and notions to HMA.

Haptic feedback interfaces have been extensively studied thanks to their relative ease of integration with robotic systems^103^. For example, electrotactile feedback has been used to transmit the amplitude of the myoelectric signal generated by the subject to control a prosthesis, allowing better grasp performance, compared to traditional force feedback^104^. Moreover, electrotactile stimulation can produce highly intuitive feedbacks with high spatial resolution^105,106^ that can be implemented to improve closed-loop control^107,108^. Despite this potential, some drawbacks of electrotactile stimulation need to be considered, notably: (i) the need to calibrate the stimulation intensity according to subject sensitivity threshold to avoid painful or annoying stimuli, and (ii) the trade-off between number of channels and calibration time.

Vibrotactile feedback has also been successfully used to carry information regarding prosthesis state, contact with objects and force^30^, and it has also been exploited to induce kinaesthetic illusions. Indeed, using a vibrotactile stimulator to apply an 80 Hz vibration to a muscle tendon entrains muscle spindles’ Ia fibres and induces an illusory movement^109–111^. A few studies have induced kinaesthetic illusions to provide real time feedback of a robotic hand, both in healthy participants^57^ and in amputees^112^. Feedback exploiting kinaesthetic illusions has the advantage to relay SRL postural information with the same sensory modality between source and user, thus the brain can access it without translating it first^113^, lowering cognitive burden. However, kinaesthetic illusion techniques require a rigid experimental paradigm, can easily interfere with natural proprioceptive sensations, and are subjected to habituation or may eventually become annoying. The possibility of exploiting different feedback sub-modalities within the realm of haptics (e.g., a combination of kinesthetic illusions and vibrotactile stimulation) to convey a rich and diverse SRL feedback should be further investigated and could be a particularly promising approach.

A pivotal aspect to discuss when considering haptic feedback is the body location where it is delivered (Fig. 1, right). Location impacts reaction times in response to tactile stimulation; for instance, it was shown that knee flexion/extension response to tactor stimuli presented at the leg were roughly 60 ms slower compared to elbow flexion/extension response to arm tactile stimulation^114^. Different sensitivity to tactile stimulation needs to be accounted for as well: due to different density of skin receptors and afferent nerve fibres, spatial acuity, measured through two-point discrimination threshold (TPDT), shows the greatest resolution on fingertips and the worst on the back^115,116^. However, when choosing which body part to stimulate, wearability and usability of the system also come into play, so several haptic feedback systems have been designed as a waist or belt solutions^117,118^, considering the large skin surface available and the specific application needs. In the context of HMA, the body location where haptic feedback is delivered depends on task features, SRL type and information bandwidth. Studies on SRL have used the contralateral fingers^20,119,120^ and the back of the hand^121^. Relaying SRL feedback on the upper limbs, however, has an important drawback since it could interfere with natural proprioception of a body part that could be used concurrently (e.g., tri-manual manipulation or three-tools surgical assistance). For this reason, several authors preferred to place haptic stimulators on the lower back^122^, feet soles^123,124^ or legs^84,125,126^, since previous results demonstrated that complex haptic stimulations can be successfully delivered to lower limbs as well^127–129^.

In addition to haptics, auditory feedback could be employed, with sonification representing a particularly interesting option. Sonification consists in translating certain physical quantity (e.g. force applied by a pencil on a surface) into a sound whose intensity or frequency can vary accordingly to the considered parameter^130–132^. Despite never being tested in a proper SRL protocol, sonification was shown to improve motor learning during a simple key pressing task^131–134^, and recalibrated perception of body size, such as height^135^ and finger length^136^. In prosthetics, information about prosthesis configuration delivered though sound cues can improve control performance and reduce cognitive burden^137,138^. The possibility to convey complex sensorimotor information through sonification represents a non-invasive option that fits well into the HMA scenario, especially if sounds are used to code parameters that cannot be easily assessed with incidental feedback (e.g., contact force between SRL and environment) or specific critical events (e.g., reaching the maximum torque that can be applied to a joint).

The possibility to use alarm signals to mark salient events using a dedicated stimulation modality (e.g. electrotactile stimulation) has already been proven in prostheses^139,140^ and it very-well fits SRLs, which are capable of exerting higher force than a human being, and can move in a workspace which partially overlaps with the users’ one. Hence, clearly signalling when certain limits are reached by the SRL becomes important to preserve humans’ safety and environment or objects’ integrity.

### Usefulness and efficacy of supplementary feedback for SRL control

Conveying supplementary information about the SRL to the user poses all the typical challenges of artificial feedback, such as (i) finding the right strategy to code artificial sensory information into a language that can be understood by the nervous system; (ii) maximizing the amount of information that can be streamed through a certain sensory channel in a specific time window (i.e., bandwidth), by translating only the parameters which better define the SRL status; (iii) finding a satisfactory trade-off between richness of delivered information and cognitive burden. Moreover, supplementary SRL feedback poses a series of further constraints related to the coexistence of supplemental feedback on top of natural one: the brain must decode SRL feedback without neglecting or interfering with sensory information coming from the natural body, and, contrarily to prosthetics, there are no residual neural resources that can be repurposed for conveying feedback.

The usefulness of sensory feedback in achieving better performance is heavily influenced by the level of experience and familiarity the user has with that specific feedback, as the relationship between internal models and feedback changes^12^. Indeed, low performance at the very beginning of a learning process is partly due to the lack of internal models^30^, which are continuously improved by sensory cues^68,141^. After extensive training, optimized internal models could allow participants to rely on inverse models for implementing feedforward controls, reducing the need for sensory feedback^107^. A very informative yet unintuitive sensory cue (e.g., SRL joint torque) might require longer training before it can be learned and produce its benefits^84^, while a supplementary feedback that exploits spontaneous associations might drastically reduce the need for training^142^. Hence, learning time should be a main aspect to consider when comparing the efficacy of different feedbacks.

When supplying additional sensory feedback, it is important to also take into account its relationship with other sources of feedback present at the same time. Considering the limited bandwidth, the ideal supplemental feedback should provide sensory information which is not available through any other sensory gateway. On the other hand, redundancy of information (e.g., vibrotactile stimulation coding the same information as vision) might still improve performance thanks to sensory integration^122^, especially if the different feedback signals possess independent noise. However, in this case bandwidth will not be optimized and the additional value of supplemental feedback would be modest.

A Bayesian approach to multisensory integration^44^ also suggests that to isolate and test the specific contribution of a supplemental feedback, the reliability of other feedbacks signals should be reduced by decreasing their quality (e.g., blurring the visual feedback), thus forcing the user to rely on the supplemental feedback.

Finally, determining which parameter and stimulation pattern will maximize feedback usefulness is crucial. For example, a wearable sixth-robotic finger was employed to complete a pick-and-place task^143^ and force feedback was delivered either through: (i) vibration bursts that signalled contact with the grasped object and actuators’ force limit; (ii) vibration bursts that signalled the intensity of the force exerted at discrete intervals; (iii) continuous vibrations proportional to the intensity of the force exerted. Even though participants subjectively reported that signalling contact and force limit was the most effective strategy, different codings lead to similar performance, which was always better than the no feedback condition, possibly because discrete information was sufficient to discriminate contact. The authors attribute these results to the possible saturation of skin receptors after continuous stimulation and the excessive richness of feedback information conveyed, stressing the importance of not overloading participants on a low-level and on a high level sensory processing, especially if the user did not train enough to build an intuitive decoding framework for sensory feedback. Taking into account both the cognitive load and tasks requirements, a simple corrective feedback which guides participants in minimizing an error (e.g., directional vibration proportional to the distance from a target) could lead to better results compared to more complex continuous tactile feedback which signals the absolute status of the device^127^. This is further supported by the fact that when continuous and discrete feedback^139,140^ are combined together, discrete signals dominate over continuous one^144^.

Besides accuracy, delay can determine the usefulness of sensory feedback. This is true for motor control in general^71,145^ but becomes particularly relevant for HMA because SRL sensorimotor loop implies longer delays than physiological limb control due to encoding of the information, delivering through mechanical devices, and subject’s decoding of an unnatural pattern. Latency could negatively impact SRL control performance, and even impair learning (e.g., attenuation of learning rates to adapt to visuomotor perturbation was shown when visual feedback was delayed by 200 ms)^146^. Some of these delays could be reduced to a minimum by employing appropriate hardware and software and by exploiting fast sensory modalities (e.g., somatosensory feedback), but most of it can hardly be mitigated. However, in a recent study on sensory prediction, it was shown that systematic sensory delays can rapidly be learned and attenuated by the brain^147^. Thus, to maximise supplementary feedback usefulness in HMA and possibly mitigate the delay issue, SRL users could be exposed to the specific, inevitable delay and only once their internal models have rearranged to account for it, engage them into SRL control learning.

### Impact of feedback information on control of an SRL

Afferent information coming from the SRL can be conveyed to the user through either cross-modal or homo-modal sensory stimulation. For instance, SRL proprioceptive information, such as the end-effector position, can be conveyed using vibrotactile stimulators (i.e. cross-submodal)^84^; on the other side, tactile information as the end-effector contact force measured by a force sensor can be conveyed by applying a pressure on participants body (i.e. homo-submodal)^143^. Interestingly, different studies employing both homo-modal and cross-modal stimulation strategies obtained improvements in the parameters which better describe human behaviour and motor control: force regulation, accuracy and task completion time^148^. The results of the studies investigating SRL supplementary feedback are discussed below and resumed in Table 1.

#### Improvement in force regulation

Being able to correctly regulate grip and load force allows us to exert the minimum amount of force required to complete a task without wasting energy, and above all, without risking injuries and collateral damages to manipulated objects. Indeed, this ability becomes handy in several scenarios, from daily life activities (e.g. lifting a fragile object with a force level sufficient to prevent slips but not so much to crush it)^149^ to surgical tele-operations^95,103,150^, where a misuse of force could potentially cause severe injury to the patient. In employing a tool, such as an SRL, that could be capable of exerting a force greater than a human limb, proper force regulation is crucial as well. Sensory feedback improves force regulation in prosthesis control^31^, and the same is reported by other studies when controlling different types of SRL. For instance, in an experiment designed to minimize the role of visual feedback while using a wearable SRL, conveying end-effector force by applying proportional pressure against the participant’s lower back allowed to match a desired target force^122^. In another study, a wearable sixth robotic finger was employed to augment human manipulation capabilities. The SRL was controlled through a ring interface worn on the index finger, and haptic feedback, in terms of force exerted and object contact, was delivered through vibrotactile stimulator placed on the ring^143^. Vibrotactile stimulation could either be: (i) continuous and proportional (frequency and amplitude) to the force exerted by the robotic finger when handling an object; (ii) delivered in bursts, to signal force thresholds; (iii) delivered in bursts, to signal the making/breaking contact with it. During a pick and place task, with any of the three feedback types, participants were able to complete the task by exerting roughly half of the force employed when doing the task without SRL supplementary feedback. Coherently, participants reported that they considered the feedback to be an effective source of information regarding the object interaction. Furthermore, in a different study, a platform made of two non-wearable robotic arms was employed^124^ to manipulate objects in conjunction with participant’s natural arms. Each robotic arm was controlled through a foot-guided ipsilateral platform moving in Cartesian space, which relayed haptic feedback to the user in three DoFs. Participants were able to modulate force exerted with the robot to successfully lift and manipulate an object and even compensate for abrupt disturbances (i.e., vibrations caused by hammering on the workspace).

#### Improvement in accuracy

Accuracy, considered here as the ability to successfully complete a task (e.g., reaching or pick-and-place) by making as few mistakes as possible, is another parameter usually adopted to estimate participants’ performance. In a two-conditions experimental design differing only for the employed feedback, accuracy becomes a reliable indicator of its quality and functionality. This has been done in a pointing task, where participants who received only electrotactile feedback, after a long, multi-session training, exhibited greater accuracy then the ones who underwent the same training while receiving both electrotactile and visual feedback^20^.

An intriguing feedback strategy allowed participant to improve their accuracy in a task requiring them to reach and contact the tip of each finger with a wearable robotic extra-thumb controlled through the vestigial posterior auricular muscle^121^. Two vibrotactile stimulators, placed on the back of the contralateral hand at the base of the little and index fingers, were used to induce a single phantom tactile sensation perceived between them. The vibration intensities were modulated according to the joint angle of the robotic thumb to make participants perceiving the tactile illusion at the base of the finger toward which the SRL was moving: the higher the intensity of a given stimulator, the closer to that stimulator the sensation was perceived^151,152^.

Vibrotactile stimulation carrying the SRL end-effector position, in terms of Cartesian space, allowed participants to complete an SRL end-effector tracking task with higher accuracy compared to a non-informative feedback signal, both offline^84^ and online^126,153^.

Increase of accuracy due to haptic artificial feedback has been reported also when full visual feedback was available; feedback of the force at the tip of a wearable robotic extra-thumb delivered with electrotactile stimulation of the thenar eminence resulted in lower error rate in a bolt-picking task^120^.

Finally, the impact of force feedback of a wearable robotic sixth finger has been studied in chronic stroke patients performing activities of daily living^119^. Using the SRL improved patients’ dexterity and performance in two out of five tested activities, but -despite patients subjectively preferred the haptic feedback condition- the improvement was feedback-independent.

#### Improvement in completion time

In most of the applications envisioned for SRLs (e.g., acting in hazardous environment, handling dangerous materials, rescuing people, performing robotic surgery), beside the achieved level of accuracy, the ability to complete a task quickly is essential. However, only a few studies have investigated possible advantages of SRL sensory feedback in completion time: it has been shown that adding haptic feedback to vision allows to complete a pick-and-place task in almost half the time^120,143^, compared to a condition without supplementary feedback. Finally, it was proven that participants are faster in replicating the position of an SRL end effector when they are provided Cartesian space feedback, compared to joint torque feedback or to a non-informative signal^84^.

## Sensory feedback and SRL embodiment

Embodiment is a multifaceted concept whose definition can vary greatly depending on the field of study. Indeed, its application field can range from social psychology, where it corresponds to the assumption that mental states are grounded into bodily states^154^, to neuroengineering, which considers hard embodiment as the mimicking of biological functions^155^. Here we define embodiment in relation to human augmentation: an SRL is embodied when it is included in the body representation, so that ownership and agency^156,157^ are experienced. When considering human augmentation, it is also important not to hinder the embodiment of natural body parts.

Several qualities and constraints of embodiment, which have been largely investigated in cognitive neuroscience and more specifically in tool use, could be translated to HMA. Indeed, it is known that everyday tool use can modulate peripersonal space and change natural arm kinematics^158,159^. Similarly, people can judge where on a tool a tactile stimulus is applied with surprising accuracy, despite the complete absence of afferent signals coming from the tool^160^. These effects are usually considered implicit marks of embodiment, related to the inclusion of the tool in the body schema^161^, and could be extended to SRLs as well. However, in tool use not all the multifaced aspects of embodiment are always affected. For instance, in many situations we use tools precisely because it is unsafe or unsanitary to use our actual body, for example when stirring a pot of soup. In such cases, we can benefit from transparent control of the tool which typically comes with tool embodiment, but it is imperative that we clearly distinguish the tool from our actual body. On the other side, in many HMA applications, SRLs are not meant to be mere tools, but more an extension of the human body, and their inclusion into mental body representations can be considered as a pivotal objective.

Neuropsychological studies have identified a range of different mental body representations relating to different aspects of sensorimotor function^162,163^. For example, in their classic study, Head and Holmes^164^ distinguished a body schema tracking real-time changes in body posture from a superficial schema mediating tactile localisation on the skin. Other studies have implicated representations of body semantics^165^, body structure^166^, and the body’s metric properties^167^. The most widely investigated distinction, however, is between body image and body schema. While the former is a perceptual representation underlying our conscious experience of our body^168^ the latter is a sensorimotor representation exploited for guiding action^41^ and represents the most interesting form of body representation in the context of HMA. Indeed, no proficient action is possible without the knowledge of relevant body parameters (e.g., limb weight, length, degrees of freedom, joint stiffness), and the same is likely true for SRLs.

### Embodiment beyond natural human morphology

A first important aspect of embodiment that must be addressed in the framework of HMA is whether it is feasible or not to include supernumerary limb into our body schema. The original version of the rubber hand illusion^55^ required the subject’s real hand to be out of view in order to embody the fake hand. A modification of the experimental paradigm in which the real hand remained visible demonstrated that healthy individuals can experience having three arms, all capable of sensing touch applied to them and perceived as being part of the participant’s body, as assessed through questionnaires and skin conductance response to a threatening stimulus^169–171^. This phenomenon, which violates the usual body morphology, could be explained according to a probabilistic framework^172^. When real and fake hands are synchronously stimulated in plain sight, the same visuotactile stimulation is coming from two different, but close, points in space. Instead of taking side, the brain builds a bimodal probability distribution, accepting that the hand can be located in two different places at the same time^173^ and generating the feeling of having three hands in total.

The experience of supernumerary body parts can also be generated with visuo-tactile illusory matches induced using a mirror box. When the hand behind the mirror is touched while they see touch being applied to empty space next to their hand, questionnaires show that people report experiencing a supernumerary sixth finger being touched^174,175^. When the visual stimulus moves along a distance bigger or smaller than the tactile stimulus, people experience supernumerary fingers of different lengths^176^, with no change to the perceived length of the five actual fingers. This shows that the supernumerary finger is not just a copy of an existing one, but a distinct body part owning independent features.

The limit to how many additional limbs can be embodied has been pushed even further^177,178^, as two supernumerary limbs can be assimilated in the body image^178^, especially under strong facilitating conditions: all four hands moved in the same way and received congruent tactile stimulation, and they had the same appearance as the real hand^179^. However, this change of body representation only affected body image while leaving body schema unmodified, as shown by movement kinematics in a reaching task^178^.

Finally, a high degree of anthropomorphism, despite being helpful, is not necessary for inducing embodiment as proven by a large amount of scientific literature in which ownership can be felt for rubber^180^, robotic^57^ and virtual^181,182^ hands, robotic prostheses^35^, stuffed gloves^183^, morphologically different hands^184^ and even wooden objects^185,186^, as assessed mainly through questionnaires, proprioceptive drift and skin conductance responses to threats. Some studies used these tools in addition to body kinematic analysis to show that even tails^187^ and wings^188^ can be embodied by participants able to control them, albeit not reaching the same levels of embodiment of more human-like limbs. This curious finding is dramatically interesting for HMA as SRL can sometimes be not completely anthropomorphic: for example, the use of a SRL with more degrees of freedom than a human limb may be desirable.

Most importantly, it has been reported that embodiment over additional limbs did not determine a disembodiment of the natural one^171^.

In addition to subjective feeling of embodiment reported by participants, objective measures of supernumerary limbs embodiment have been reported as well. For example, participants showed skin-conductance responses when the supernumerary hand was threatened^171^. Moreover, merely observing a realistic artificial arm, located in a plausible position and orientation and physically connected to the body, led participants to perceive it as a third arm belonging to their body. This feeling of embodiment positively correlated with shifts in the topography of the somatosensory homunculus: higher level of embodiment corresponded to a more medial-superior position of the thumb representation, as if the existing representation was leaving some space for the newly embodied arm^189^.

It is important to note that different methods for assessing embodiment of SRLs possess different potentials. Indeed, while explicit measures (e.g., questionnaires) are easy to implement and provide straightforward results, they can be influenced by participants’ susceptibility, and given the peculiarity of experiencing supernumerary limbs, they might fail to highlight subtle changes in SRLs embodiment. On the other hand, some implicit behavioural measures, such as proprioceptive drift, are known to not always correlate with perceived ownership^190^, hence their reliability can sometimes be questionable. While a complete overview of embodiment assessment methods is out of the scope of this work, we deem that their choice should be considered carefully.

### Benefits of SRL embodiment

Embodiment is known to be generated by congruent multisensory feedback^53,54^: a lifetime of body-related sensory cues bound by spatiotemporal congruency proves us that our body is indeed our own and we exert a certain level of control on it. The connection between embodiment and sensory information is so tight that, under certain conditions, the causal-effect relation can work the other way around: once embodiment of an artificial limb has been established, it can induce the experience of phantom sensory feedback when the artificial limb is stimulated. For instance, participants reported that they could experience haptic sensations on their real hand when they observed an embodied avatar touching a curtain with its hand, despite not receiving any real haptic stimulation themselves^191^. Additionally, it was shown that once embodiment is established, participants are less prone to notice asynchronies in visuotactile stimulation (Table 2, left, point 1). In other words, when an object is embodied, the brain seems to automatically fill in any small gap in multisensory feedback concerning the interaction between user and object^59^. Indeed, when participants are responsible for a self-stimulation (i.e., they feel agency for it), and its sensory consequence is systematically delayed, they rapidly learn the delay, and can quickly compensate for it, most likely by adjusting their internal model^147^. This could be particularly useful because a certain level of delay or asynchrony between multisensory stimulation could be intrinsic and inevitable (see chapter 3.2) when integrating a SRL into a human-robot ensemble.

**Table 2:** Potential benefits of SRL embodiment and how to obtain them by acting on sensory feedback features.

Potential benefits of SRL Embodiment	Feedback strategies to facilitate SRL Embodiment
1. Mitigation of feedback discrepancies (Maselli et al.^59^; Kilteni et al.^197^)	1. Provide low-noise feedback signal (Blanke et al.^213^, Hoyet et al.,^203^; Rosa et al.,^202^; Arai et al., ^200^)
2. Improved SRL motor control (Ehrsson et al.^192^; Ehrsson et al.^193^; Tsakiris et al.^194^; Grechuta et al.^195^; Schiefer et al.^196^; Rognini et al.^34^; Zollo et al.^32^; Di Pino et al.^35^)	2. Provide ecologic and realistic feedback (Fröhner et al.,^204^; Richard et al.,^205^)
	3. Exploit visuomotor correlations (Sanchez-Vives et al.^209^; Kokknara et al.^210^; Pinardi et al.^57^; Romano et al.^211^; Tsakiris et al.^212^)
3. Improvement of human-robot global kinematics (Kilteni et al. ^197^; Nishio et al.^198^)	4. Exploit visual feedback in first person perspective (Slater et al. ^214^)
	5. Provide feedback-driven gradual prior modification (Toet et al. ^215^)

References reported here do not necessarily investigate SRLs directly but provide precious insights that justify the entry they refer to.

The reciprocal influence between embodiment and sensory feedback suggests that embodiment itself could improve motor control (Table 2, left, point 2). The impact of embodiment on task dexterity and performance has yet to be determined in a real scenario, but there are several observations that support this hypothesis. Indeed, several brain areas involved in generating body ownership are also key motor control stations, namely premotor cortices, temporal parietal junction and insula^192–194^. Accordingly, a study carried out in virtual reality has shown that increasing ownership of a virtual limb decreased reaction times in a sensorimotor task^195^. Finally, bidirectional prostheses that can provide sensory feedback have been shown to boost not only movement control but embodiment as well^32,34,35,196^, corroborating their potential link.

Furthermore, it was shown that embodiment of virtual bodies or robots induced behavioural changes in users dictated by the affordances of artificial bodies. In other words, the embodied avatar shapes the way we behave, from small kinematic adjustments directly mirroring the avatar features, (e.g., slowed movement that matches the robot limited velocity)^197^ to semantic-related changes, such as higher movement variability when playing a drum if a dark skinned avatar is embodied, compared to a light skinned one^198^. The same impact could be expected when embodying an SRL, and modification of real limb kinematics, balance or simply standing posture could improve the human-robot interaction (Table 2, left, point 3).

### Methods for inducing SRL embodiment

Despite its promising potential and theoretical feasibility, research is still struggling to induce SRLs embodiment. In a recent study^123^, participants performed a pointing task with a wearable robotic arm relaying force feedback to participants’ feet soles. Interestingly, participants verbally reported that embodiment increased as the experimental session progressed. The effect size was only moderate, but this could be due to the non-anthropomorphic end-effector of the SRL. In another study^199^, participants worn an EMG-controlled sixth finger to perform a tapping task and received haptic feedback on the side of the palm, informing them of the artificial finger movement. After performing the task for about 50 minutes, participants did not perceive ownership of the supernumerary finger, even if they could actively control it, but reported significantly higher agency compared to a no-control condition. Additionally, the authors found clear correlations between reported ownership (questionnaire) and changes in body image, assessed through a finger localization task. Despite the lack of significant SRL ownership, results suggest that it could be achieved, but it would be more difficult to induce compared to a substitutive limb (e.g., rubber hand) and it would require longer period of interaction with the artificial limb. However, the role of feedback should be carefully considered: the very limited and simplified supplementary haptic feedback provided to participants might have played at least a partial role in determining the lack of perceived ownership.

Indeed, in a recent study^200^ participants performed a ball-touching task by controlling a virtual SRL through a foot interface, and could receive vibrotactile feedback on the foot, coding the contact of the SRL’s end effector with the virtual ball. Despite the relative short task (~16 min) results show both an explicit (i.e., questionnaire) and implicit (i.e., Crossmodal Congruency Task) increase in embodiment after performing the task. These results could be justified by the rich feedback provided: the contact between the back/palm of the SRL’s end effector was mapped on the foot by stimulating the upper side/sole of the foot, and the vibration used a frequency band (200hz) that can be easily and reliably detected by humans^201^. Overall, recent works concerning virtual supernumerary limbs^200,202,203^ that include facilitating factors such as first-person perspective and low-delay visuomotor integration, suggest that embodiment of SRL is more easily boosted in a virtual environment compared to a real one. Since this is most likely due to the high-quality multisensory integration that can be obtained in the former, it testifies the importance of a well-designed low-noise supplementary feedback (Table 2, right, point 1). Furthermore, virtual reality studies showed that an ecologic and realistic feedback (e.g., pressure applied on participant’s fingers to simulate the contact with a surface) is more effective in inducing embodiment of a virtual effector compared to more symbolic feedback (e.g., vibrotactile stimulation that signals contact)^204,205^ (Table 2, right, point 2).

To better understand how to successfully induce SRL embodiment, it is useful to consider the computational mechanism behind the rubber hand illusion^55^, where the ownership of the artificial hand is the best explanation the brain can give for a tactile feedback which does not match with visual feedback from the real hand, but rather with the visual feedback from the fake one. However, the brain has to mediate conflict not only among sensory feedbacks (i.e., likelihood), but also between them and the experience-related knowledge (i.e., prior)^206,207^. This latter conflict is stronger in the case of supernumerary limbs compared to RHI. Indeed, to embody them, the brain must overwrite not only the knowledge about the visual aspect of our hand (e.g., not rubbery) and its perceived position (e.g., located where our proprioception suggests)^170,179,208^, but also the knowledge about limb numerosity (e.g., having only two hands).

To make this accommodation easier (i.e., facilitate embodiment) it is possible to act on the likelihood, by using all possible strategies to empower it, or on the prior, by inducing preliminary changes to body representation. Concerning the first possibility, it is known that visuomotor correlations are a powerful tool to induce embodiment^57,209,210^ as they involve not only the perceived but also the predicted feedback, thanks to the expectation provided by the efference copy (Table 2, right, point 3). Indeed, congruency between those two feedback signals means that the effector is executing the user’s motor command, and this alone can induce the feeling of embodiment^57,211^, particularly concerning agency. To confirm the efficacy of visuomotor correlations, it was shown that their effect on body schema can propagate across limbs: visuomotor stimulation involving a single digit produced proprioceptive drift of the entire hand, contrarily to visuotactile stimulation, whose effects are digit-specific^212^. Hence, it would be useful to include an efferent component when planning an experiment to promote the embodiment of an SRL. The ideal experimental setup would require the participant to command the SRL while performing an interactive task, relying on a rich but easily understandable supplementary feedback to successfully complete the task.

Finally, if the task involving the SRL is designed to be done in virtual reality, the first-person perspective should be considered (Table 2, right, point 4). Indeed, while embodiment can be perceived also for third-person avatars, thanks to congruent multisensory stimulation^213^, it was shown that the simple adoption of a first-person perspective induces a sense of embodiment of a virtual body, without any kind of supplemental stimulation^214^.

Regarding the possibility to act on the prior, it has been hypothesized that sensory inconsistencies can be integrated more easily in the body representation if they are introduced gradually^215^. Hence, the embodiment of the SRL could be obtained through a step-by-step process, so that the posterior probability of each step becomes the prior for the following one (Table 2, right, point 5). The first step could be to embody limbs that present a low degree of conflict with the existing prior, as it happens in the classic RHI. Successively, the paradigm could shift to the embodiment of one supernumerary realistic limb, to finally focus on the proper SRL.

## Discussion and conclusion

SRL have the potential to improve abilities of healthy humans^6,10^ and induce changes in body representation (i.e., embodiment). However, to obtain an effective human movement augmentation (HMA), SRL and user must be part of a closed loop system. In this work we systematically addressed issues and potentials of supplementary sensory feedback for SRL in terms of motor control and embodiment and we proposed a novel nomenclature to clarify augmentation related terminology for feedback design.

Investigations done so far on SRL supplementary feedback highlighted improvement in three key areas of SRL control: force regulation^122^, accuracy^121,143^ and task completion time^120^. Tactile and proprioceptive feedback represent a promising feedback choice, but observations on feedback features in HMA are largely based on neuroscientific results from other fields (e.g., prosthetics), as a systematic investigation on supplementary feedback features in the framework of HMA is missing.

**Fig. 2:**
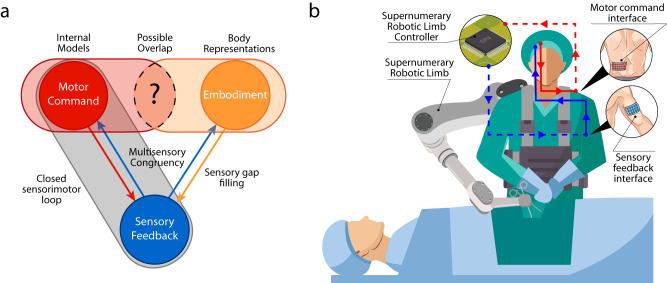
Hypothetical closed loop in human augmentation. **a** Relation between Internal Models, Body Representations and Sensory Feedback. The area with a question mark included in dotted lines represents the possible overlap between Internal Models and Body Representations that should be investigated further in future studies. **b** Example of SRL use in a surgical operation. SRL sensory information is recorded by sensors embedded on the robot, encoded by the SRL controller unit and relayed to a sensory feedback interface (dotted blue line). The sensory feedback interface stimulates the user, thus providing supplementary sensory feedback on the SRL status (solid blue line). The user produces a motor output (e.g., EMG activity) that is detected by the motor command interface (solid red line). The motor command interface relays the signal to the SRL controller unit (dashed red line) to command the robot.

Supplementary feedback should be tailored on the specific task considered and should provide reliable, high-fidelity information not available through incidental feedback^44^. Delays should be kept to a minimum, especially if they are not consistent. Cognitive and sensory overload should be avoided (i.e., discrete over continuous feedback), or at least mitigated through appropriate training, which triggers update of internal models, thus improving feedback decoding^30^. Visual modality should be avoided in favour of other unobtrusive modalities, such as somatosensation, and the stimulation should be delivered to body part with a good trade-off between spatial resolution and convenience. Finally, supplementary feedback should not interfere with natural one. These principles should be carefully considered when implementing sensory feedback in the human-robot loop (Fig. 2).

Studies on bodily illusions support the feasibility of SRL embodiment: participants can embody up to four limbs^179^, without disembodiment of real body parts, withstanding the low anthropomorphism. However, similarly to SRL feedback features, a systematic study of SRL embodiment is missing as well.

While studying the specific efficacy and usefulness of feedback features is an important first step, we deem that a concrete advancement in HMA is possible only by considering motor control and embodiment together, within the same theoretical framework. Indeed, their reciprocal influence, the common computational basis (i.e., Bayesian multisensory integration) and strong reliance on sensory feedback, suggest a likely partial overlap between the concepts of body representation and internal models (Fig. 2a), which could strongly impact not only HMA, but the study of human sensorimotor system in general.

If we consider an hypothetical closed loop involving user and SRL (Fig. 2b), the feedback’s function would be twofold: (i) modify the user’s body representation to include the SRL (i.e., embodiment) and (ii) constitute an element of comparison for the sensory feedback predicted thanks to the efference copy, to implement an accurate control policy, thus leading to a better motor control of the SRL.

As a result of the improved motor command, perceived sensory feedback would match the predicted one to a higher degree and would increase the congruency of multisensory stimulation (especially concerning visuomotor cues), ultimately leading to higher embodiment.

A boost in embodiment would improve the user experience, by making slight feedback inconsistencies less noticeable. This mechanism would generate a virtuous circle in which each component (i.e., sensory feedback, body representation and motor command) has a beneficial effect on another.

However, the definition of a detailed model should be pursued with ad-hoc experiments investigating specifically the mutual interaction between feedback-induced SRL embodiment and motor control through longitudinal training sessions. Indeed, richer feedback would require a longer training before users can adapt their internal model and successfully decode and employ the feedback. This operational constraint fits well with requirements of SRL real life application: users of SRL would most likely undergo a long and intense training before employing an SRL for precise activities (e.g., robotic surgery) or dangerous activities (e.g., handling hazardous materials). Despite that, almost all current works who studied the use of sensorized SRLs did so with remarkably short training sessions (i.e., minutes or hours). We reckon that a shift toward longitudinal paradigms, where participants undergo longer training (i.e., weeks), could provide additional and novel insights on HMA. For instance, participants could first be exposed to the SRL feedback while the robotic limb is visible, to link feedback content and stimulation patterns. After repeated sessions, whose number and duration would be determined by the complexity of the supplementary feedback provided, control of the SRL would be introduced while visual feedback would be gradually removed. Ideally, daily sessions of 2-hours duration would avoid excessive fatigue while allowing enough time to learn a decoding framework. Later, the SRL should be controlled to complete a trimanual task leveraging on supplementary feedback to supply information when visual feedback is absent. At this stage, evaluation of motor control performance with and without supplementary feedback should be carried out. Embodiment should be assessed across all sessions, to highlight its longitudinal modulation. Performing longitudinal studies in which all feedback features presented in this manuscript are systematically investigated would allow to determine how SRL motor control and embodiment evolve in time, to quantify their reciprocal influence and to clarify the specific contribution of each feedback feature. This could lead to the definition of a detailed working model that can successfully predict control performance and embodiment based on feedback features, which is presently missing in human augmentation literature.

Finally, future studies should also consider the subjective or affective experience of participants controlling and especially embodying SRLs. For instance, cosmetic appearance of prostheses is known to influence the acceptance of the device^216^, and a similar consideration can be made for SRLs. Despite that, most of them (excluding supernumerary fingers) do not possess a human-like appearance. While this does not completely prevent embodiment it might impact the subjective experience and thus human augmentation performance.
